# American Congress of Tuberculosis

**Published:** 1902-05

**Authors:** 


### Society Notes.

### American Congress of Tuberculosis.—Session of May 14, 15 and 16, 1902.

Office of the Secretary,
39 Broadway, N. Y.

New York, April 7, 1902.

*Dr. F. E. Daniel, Editor Texas Medical Journal.*

My Dear Sir: I have the honor to enclose you a copy of a letter by me received from the State Department of the government of the United States, assuring us of its kind sympathy and support with the governments of Mexico and the Central and South American Republics, as to representation at the approaching session of the congress in May next. The letter is as follows:

Department of State,
Washington, April 4, 1902.

*Clark Bell, Esq., Secretary of the American Congress of Tuberculosis, 39 Broadway, New York City.*

Sir: I have to acknowledge the receipt of your letters of the 25th and 28th ultimo, in regard to the session of the American Congress of Tuberculosis, which is to be held at the Majestic Hotel in New York City in May next.

In compliance with the request which you make, the Ambassador to Mexico, and the Ministers to the Central and South American States, have been instructed to express to those governments the pleasure with which that of the United States would learn that they found it convenient to be represented in the congress.

I am, sir,

Your obedient servant,

David J. Hill,
Assistant Secretary.

I also take pleasure in informing you that the Earl of Minto, Governor-General of the Dominion of Canada, has accepted the position of Honorary Vice-President of the American Congress of Tuberculosis and has written that that government will be represented by delegates.

I am also advised by the Hon. Sir Oliver Mowat, G. C. M. G., Lieutenant-Governor of the Province of Ontario, an Honorary Vice-President of the Congress, that steps have been taken to send representatives of the provincial government of that Province, and Lieutenant-Governor Hon. A. E. Borget, of the Northwestern Provinces of the Dominion, has sent a very strong letter of sympathy for the success of the congress of which he is an honorary vice-president.

Please announce this in your valuable journal.

I remain, sir,

Ever faithfully yours,

CLARK BELL,
Secretary.

